# Entrepreneurship education shapes creativity among Egyptian hospitality students through entrepreneurial attitudes and intentions

**DOI:** 10.1038/s41598-026-59670-w

**Published:** 2026-07-07

**Authors:** Omar Alsetoohy, Mahmoud Abou Kamar, Ahmad Samed Al-Adwan, Sayed M. Ismail, Anwar Hammad Al-Rashidi, Khaled Ahmed Abdel-Al Ibrahim

**Affiliations:** 1 Department of Hotel Management, Faculty of Tourism and Hotels, University of Sadat City, Sadat City, 32897 Egypt; 2 Business Technology Department, Al-Ahliyya Amman University, Amman, Jordan; 3 English Department, College of Sciences and Humanities, Prince Sattam Bin Abdulaziz University, Al-Kharj, Saudi Arabia; 4 Department of Psychology, College of Education, Prince Sattam Bin Abdulaziz University, Al-Kharj, Saudi Arabia

**Keywords:** Entrepreneurship education, Creativity, Entrepreneurial attitudes, Intentions, TPB, CTC, Hospitality, Business and management, Business and management, Education, Psychology, Psychology

## Abstract

This study developed and tested a psychological model explaining associations between entrepreneurship education (EE) and student creativity and entrepreneurial behavior, drawing on the theory of planned behavior (TPB) and the componential theory of creativity (CTC). Using a cross-sectional survey of 335 hospitality students and analyzing the data with partial least squares structural equation modeling (PLS-SEM), the study examined the relationships of EE with individual creativity, university-level creativity, entrepreneurial attitudes, and entrepreneurial intentions. The results show that EE is positively associated with students’ entrepreneurial intentions, attitudes, and individual creativity. Entrepreneurial attitudes and intentions appear to act as key psychological mediators in several pathways, suggesting an interconnected nature of creativity and entrepreneurial behavior. However, entrepreneurial intentions do not mediate the relationship between EE and university-level creativity. The findings provide contextual support and possible extensions to TPB and CTC by highlighting how educational interventions relate to both creative capacity and entrepreneurial tendencies in resource-constrained higher education contexts. Practically, the study provides preliminary guidance for enhancing entrepreneurship curricula and designing supportive ecosystems that promote creativity, experiential learning, mentorship, and entrepreneurial behavior among hospitality students.

## Introduction

Entrepreneurship has emerged as a cornerstone of socio-economic development, playing a transformative role in combating unemployment and driving innovation in both developed and developing nations^1,2^. Recognizing its strategic importance, entrepreneurship education (EE) has gained prominence as a key driver of entrepreneurial skills, creativity, and innovation. Beyond equipping students with the technical knowledge to launch ventures, EE nurtures an entrepreneurial mindset marked by problem-solving, opportunity recognition, and adaptability, core competencies vital for navigating today’s dynamic global economy^3^.

Universities are pivotal in fostering entrepreneurship, serving as incubators for entrepreneurial behavior and hubs of innovation. This is particularly crucial in fragile entrepreneurial ecosystems, where cultural, institutional, and economic contexts play a decisive role in shaping outcomes^4,5^. In emerging economies like Egypt, EE has been increasingly leveraged as a strategic response to high graduate unemployment and as a catalyst for economic resilience. Initiatives such as entrepreneurship clubs, innovation commercialization offices, and government-supported accelerators aim to address institutional gaps and nurture a culture of creativity and entrepreneurship^6^.

However, significant knowledge gaps persist regarding the association between EE, entrepreneurial intentions (EI), and creativity in developing economies. While extensive literature exists on EE in developed contexts, where robust institutional support and resource-abundant ecosystems enable effective program implementation^7,8^, these findings may not fully translate to resource-constrained settings like Egypt. Here, the interplay of limited institutional capacity, cultural norms, and resource availability introduces unique complexities^3,9^. For example, EE’s potential to enhance entrepreneurial attitudes (EA) and intentions may be undermined in environments with risk-averse cultures and inconsistent pedagogical approaches^5^.

Crucially, much of the theoretical grounding in EE research, including the Theory of Planned Behavior^10^ and Amabile’s Componential Theory of Creativity^11^, has been developed within Western contexts and assumes a degree of cultural and institutional maturity. In contrast, scholars such as El Tarabishy et al.^12^, Amofah and Saladrigues^13^, and Alharbi et al.^14^ highlight the limitations of applying these theories wholesale in developing countries. For instance, in MENA and Sub-Saharan African contexts, the effects of EE on attitudes and intentions are more deeply embedded in socio-cultural factors such as risk aversion, family influence, and restricted access to capital. Similarly, Mahendra et al.^15^ and Karimi et al.^16^ argue that the association between EE and entrepreneurial behavior is moderated by environmental variables, underscoring the importance of adapting theoretical models to contextual conditions.

The inconsistency in EE delivery methods further complicates its evaluation. While some programs emphasize experiential learning, others rely on theoretical instruction, often without standardized frameworks to assess outcomes^17,18^. As a result, the metrics used to evaluate EE, ranging from entrepreneurial intention to creativity and startup success rates, yield mixed conclusions about its efficacy^2,19^. Moreover, the cultural and institutional factors influencing entrepreneurial behavior in emerging economies, including Egypt, remain underexplored^4,20^.

What is not known is if, and by what mechanisms, EE impacts student creativity in the context of an underdeveloped, risk-averse, emerging economy with scarce resources, and a low level of institutional support and cultural acceptance of entrepreneurship. This study seeks to address these gaps by examining the relationships between EE, entrepreneurial intentions, and creativity among hospitality students in Egyptian universities.

This study draws on the framework proposed by Wang et al.^21^, which examines the relationship between EE and entrepreneurial creativity through a newly articulated lens that incorporates the role of inspiration as a mediating mechanism. Wang et al.^21^ framework highlights how EE stimulates inspiration, which subsequently enhances creativity, offering a nuanced understanding of how entrepreneurial behaviors are cultivated. Their study, which surveyed 1873 students across 36 Chinese universities, underscores the importance of EE in fostering creativity at both individual and institutional levels. While Wang et al.^21^ findings provide a valuable theoretical foundation, they emerge from a context characterized by robust institutional support, resource-rich ecosystems, and a distinct cultural backdrop. These factors contrast sharply with conditions in Egypt, where institutional and financial constraints, coupled with risk-averse cultural norms, pose unique challenges for understanding the role of EE. In such a context, the mediating mechanisms proposed by Wang et al., including inspiration, entrepreneurial attitude, and intentions, may interact differently with individual and institutional creativity. By adapting and contextualizing this framework, this study explores the association between EE, creativity, and entrepreneurial intention in the Egyptian context, offering insights into the interplay of these factors within a resource-constrained environment.

The hospitality sector provides a particularly relevant context for this investigation due to its reliance on creativity, adaptability, and innovation. In an industry characterized by high competition and customer-centric demands, fostering entrepreneurial creativity among hospitality students is essential for their professional success and the sector’s development. However, the sector’s unique demands also require tailored EE approaches that integrate both theoretical knowledge and experiential learning to enhance service innovation, problem-solving, and adaptability, skills critical to maintaining a competitive edge^19^. By situating the research in Egypt, this study offers nuanced insights into the challenges and opportunities of implementing EE in an emerging economy. Specifically, the research explores:

- To examine the association between EE and students’ entrepreneurial attitude (EA) and entrepreneurial intention (EI).
- To investigate the interplay between entrepreneurial intention (EI) and individual creativity (IC) and university-level creativity (UC), alongside the direct effects of EE on creativity outcomes.
- To analyze the mediating roles of entrepreneurial attitude and entrepreneurial intention in the relationship between EE and creativity outcomes in the Egyptian higher education context.

The study focuses on the nexus of EE, attitude, intention, and creativity within the university context because these elements play a pivotal role in shaping entrepreneurial outcomes^22^. Particularly in the Egyptian context, there is a lack of research examining how these variables interact and influence business practices within a unique cultural and economic environment. Existing literature predominantly addresses entrepreneurship in Western contexts, where socio-economic and educational conditions differ significantly^23^. By focusing on these entrepreneurship practices, the study aims to identify culturally relevant insights that can inform both academic programs and policy-making.

Although the relationship between EE and entrepreneurial outcomes has been extensively studied, current research often assumes a one-size-fits-all model, overlooking cultural and contextual nuances^24^. For instance, in Egypt, where socio-economic barriers and institutional challenges are prevalent, the effectiveness of EE and its interplay with attitude, intention, and creativity remain underexplored^12^. Some studies even present conflicting findings about whether EE directly fosters business success or if it merely influences entrepreneurial intentions. This inconsistency calls for a more nuanced investigation tailored to specific contexts, which this study seeks to provide.

Some studies suggest that EE positively influences entrepreneurial intention and creativity, which in turn enhances business success^25^. However, others argue that the link between these variables is weak or context-dependent, with factors like culture, institutional support, and economic conditions significantly moderating these relationships^26^. In the university context in Egypt, this tension is amplified due to disparities in educational quality and access to resources. Resolving this tension is critical to designing more effective educational programs and interventions.

This study makes four primary contributions. First, it addresses a theoretical gap by contextualizing the TPB and CTC for emerging economies. While these frameworks have received substantial empirical support in Western contexts with mature entrepreneurial ecosystems^7,8^, their applicability to resource-constrained settings like Egypt, characterized by risk-averse cultural norms, weak institutional infrastructure, and high graduate unemployment, remains largely untested. This study explicitly incorporates macro-level (cultural norms, institutional pressures) and micro-level (resource constraints) boundary conditions to examine whether theory-based relationships are held in this distinct context.

Second, the study provides novel empirical evidence on the efficacy of EE in a resource-constrained environment. By focusing on hospitality students, a group requiring high levels of creativity and adaptability, this research identifies two mediating mechanisms (EA and EI) as well as a non-mediating mechanism (in the EE–UC link). The stratified random sample of 335 participants across six Egyptian universities bridges the literature gap dominated by data from developed contexts^5,8^.

Third, the findings offer practical recommendations for policymakers and educators seeking to design contextually relevant EE programs that balance theoretical instruction with experiential learning to address cultural barriers such as risk aversion and limited institutional support. The study highlights the importance of institutional support mechanisms, entrepreneurship clubs, innovation hubs, and accelerators as catalysts for translating EE outcomes into tangible entrepreneurial activities^5,6^.

Fourth, from an industry-specific perspective, the study demonstrates how EE can be tailored to creativity-driven sectors by examining hospitality students who inherently require high levels of creativity and adaptability. The findings suggest that strengthening entrepreneurial intentions and intentions significantly enhances individual creativity outcomes, whereas university-level creativity requires systemic support beyond individual-level interventions^19^.

Investigating these relationships within Egypt’s socio-economic landscape contributes to a deeper understanding of how EE can be leveraged to foster entrepreneurship and creativity, particularly in underexplored contexts. The following sections outline the theoretical framework, research hypotheses, and methodological approach, providing a foundation for examining the nuanced dynamics of EE in Egyptian universities.

## Theoretical framework and hypotheses development

This study is grounded in multiple interrelated theoretical perspectives that collectively inform the proposed relationships between EE, entrepreneurial intention, attitude, and creativity in the university context.

As the core theoretical foundation, the Theory of TPB^10^ posits that intention is the immediate antecedent of behavior and is influenced by three factors: attitude toward behavior, subjective norms, and perceived behavioral control. This theory serves as the central framework, widely used to explain entrepreneurial intention^27,28^. This theory supports hypotheses regarding entrepreneurial attitude and intention (H1–H4) by explaining how EE influences students’ evaluative judgments and motivational orientations toward entrepreneurship. For instance, hypotheses H1 and H3 are directly derived from TPB’s assertion that attitude and intention are functionally linked.

Recent empirical findings further confirm the usefulness of TPB in the context of EE. Zhuang et al.^29^ examined the effect of personality traits on start-up preparation among young adults in Hong Kong and found that attitudes toward entrepreneurship and perceived behavioral control significantly mediated the relationship between openness to experience and entrepreneurial preparation behaviors. These findings provide contemporary support for the core assumptions of TPB within an Asian entrepreneurship context and suggest that attitudinal factors remain significant predictors even after controlling for personality-related influences.

Similarly, a recent meta-analysis by Lv et al.^30^, based on 36 studies, revealed that TPB explains approximately 40–50% of the variance in EI across different cultural contexts, with attitude emerging as the strongest predictor in collectivist cultures, with attitude emerging as the strongest predictor in collectivist cultural settings, including contexts relevant to Egypt. In hospitality-focused samples, Aliedan et al.^31^ confirmed that TPB factors, attitude, subjective norms, and perceived behavioral control significantly mediate the relationship between university support and EI among Saudi hospitality students, thereby demonstrating the theory’s applicability to contexts comparable to the Egyptian hospitality setting examined in the present study.

Collectively, these recent findings support the generalizability of TPB while simultaneously highlighting the need for contextualization, a gap this study addresses by examining TPB within the resource-constrained Egyptian context.

Amabile’s^11^ CTC underpins the study’s treatment of creativity as both an individual trait and an organizational outcome. This theory emphasizes the importance of domain-relevant skills, creative-thinking processes, and intrinsic motivation, factors influenced by EE. The Componential Theory of Creativity^11^, supported by work such as Edwards-Schachter et al.^32^ and Runco and Pritzker^33^, informs the creativity dimension. To consider the influence of environmental and cognitive feedback loops. Hypotheses H5 to H8 are anchored in this theory, where EE is conceptualized as a driver of creativity at both individual and institutional levels.

Bandura’s Social Cognitive Theory^34^ highlights the reciprocal interactions between personal factors, behavior, and environmental influences. It provides a lens for understanding how university support structures and cultural norms influence entrepreneurial mindset and creativity, especially in emerging economies^15,16^. This theory implicitly supports the mediating hypotheses H9 and H10, especially in explaining how contextual factors shape entrepreneurial behavior and creativity.

The Entrepreneurial Event Model by Shapero and Sokol^35^ focuses on perceived desirability, feasibility, and propensity to act as critical determinants of entrepreneurial intention. This model helps explain how EE enhances students’ belief in their ability to act entrepreneurially, supporting H1 and H2. It is particularly relevant in developing economies, where external barriers influence the perceived feasibility of starting a venture. It supports the role of perceived feasibility and desirability in forming entrepreneurial intention^36,37^. By integrating these theories, this study constructs a holistic framework to examine how EE influences both the cognitive (attitude and intention) and creative (individual and institutional creativity) aspects of entrepreneurial development within the higher education context of Egypt.

**Fig. 1:**
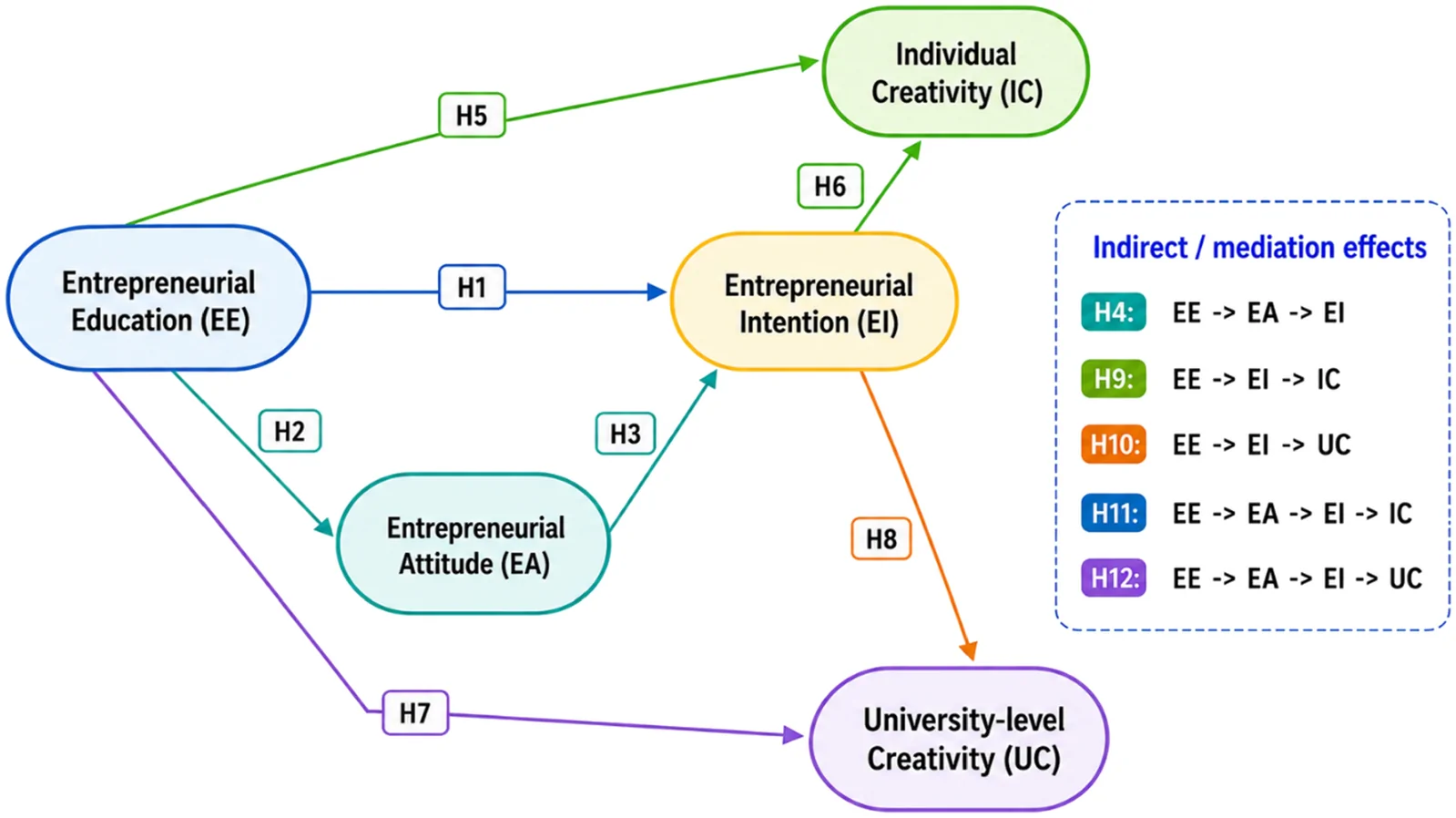
The conceptual framework. *Source*: Author’s preparation.

The proposed conceptual framework, detailed in Fig. 1, visualizes the hypothesized relationships and pathways.

### Synthesis of theoretical constructs: cross-theory mechanisms

The convergence of the TPB, CTC, SCT, and EEM examined in this paper operates through three distinct construct-level interfaces. First, the TPB–CTC interface links entrepreneurial intention (the closest behavioral antecedent in TPB) with individual creativity (the creative outcome in CTC). We propose that entrepreneurial intention may stimulate creativity through what Amabile^11^ refers to as intrinsic motivation, the internal drive underlying creative thinking. Recent evidence supports this mechanism, showing that fostering creativity is positively associated with entrepreneurship and that this relationship operates through intrinsic motivation and innovation as mediating mechanisms^38^. More specifically, students with strong EI are more likely to develop domain-relevant skills (CTC) and engage in creative-thinking processes, as entrepreneurial intention provides the goal-directed motivation necessary for creative exploration. In this way, the proposed relationship applies TPB beyond behavioral prediction by linking entrepreneurial intention with cognitive activation processes related to creativity.

Second, the SCT–CTC interface connects contextual factors, particularly university support systems, with creative outcomes. Drawing on Bandura’s^34^ concept of reciprocal determinism, students’ creative behavior is understood as both shaped by and shaping their learning environment. In this study, this relationship is operationalized through university-level creativity, which reflects institutional conditions that may either facilitate or constrain individual creative expression.

Third, the EEM–TPB interface integrates the concepts of perceived feasibility and perceived desirability from EEM with the attitude-formation process in TPB. Shapero and Sokol’s^35^ concept of perceived feasibility closely resembles perceived behavioral control in TPB, while perceived desirability aligns with entrepreneurial attitude. This integration enables us to conceptualize EE as a broader contextual mechanism that influences both feasibility perceptions, by fostering entrepreneurial skills, and desirability perceptions, by shaping entrepreneurial attitudes.

### Entrepreneurship education

Students’ entrepreneurial mindsets, defined as a set of attitudes, behaviors, and skills that enable individuals to identify opportunities, take initiative, and navigate uncertainties in pursuit of innovation, are significantly influenced by EE^39^. For instance, individuals with an entrepreneurial mindset often display traits such as resilience, creativity, and a proactive approach to problem-solving. EE provides students with valuable insights into entrepreneurial studies, helping them cultivate these attributes. As digital technology and innovation become increasingly important, the need for people with entrepreneurial abilities is rising^40,41^. Numerous studies on EE have emphasized theory, practice, and the observation of entrepreneurs’ actions and learning processes. For example, Fayolle and Gailly^22^ asserted that EE affects students’ mindset and helps them obtain knowledge related to entrepreneurship, guiding them into good career choices. Its focus is on fostering individuals who are self-employed and self-sufficient^42^. It enhances university graduates’ self-reliance and self-employment, serving as a route to work opportunities and job creation^43^. Moreover, EE contributes to the innovative capacity of an economy and fosters the development of fresh ventures and ideas^44^. However, in the case of vocational school students in Indonesia, the investigation revealed that EE had no appreciable influence on students’ entrepreneurial intentions, potentially because it emphasizes knowledge and theory rather than practical application and project-based learning^45^.

#### The association between EE and entrepreneurial intention

Academics have shown an interest in studying the effects of EE on students’ intentions to become entrepreneurs. It has been found that participating in EE programs results in a stronger desire to begin businesses and a greater conviction that one can succeed in entrepreneurship^46^. A recent study by Shah et al.^47^ compared two groups of students: one with EE completed and the other without. The findings demonstrated that students’ attitudes and intentions regarding entrepreneurship were significantly improved by EE. This is because such education provides students with the necessary abilities and expertise to initiate and oversee businesses, increasing their confidence and inspiration to take up entrepreneurial endeavors. A recent meta-analysis carried out by Lv et al.^30^ indicated that EE is positively associated with college students’ intention to launch their own companies. The analysis examined 36 studies published in the last decade and determined that there is a significant positive relationship between EE and entrepreneurial intention. The study suggests that EE not only teaches students the necessary skills and methods for launching a company but also shapes a positive perception of entrepreneurship and confidence in their ability to achieve their goals. Similarly, another study by Kusumojanto et al.^45^ reached a similar conclusion, finding that the relationship between entrepreneurial intention and education is mediated by entrepreneurial mindset. This suggests that the perspectives of students on entrepreneurship may influence the effect of EE on entrepreneurial intention. In addition to its direct and indirect effects, previous research suggests that EE may also be considered a contextual variable that strengthens or weakens the relationships between entrepreneurial predictors and outcomes at both the individual and organizational levels. Although this role remains underexplored in the literature, it has important practical implications. Mukhtar et al.^39^ investigated the relationship between the institutional environment and entrepreneurial intention (EI), with national culture serving as a moderating factor. Their study of 412 university students in Indonesia found that EE significantly strengthened the relationship between perceived institutional support and entrepreneurial intention. More specifically, the positive effect of institutional support on entrepreneurial intention was stronger among students who had participated in EE activities. These findings suggest that EE may equip students with the skills needed to identify and leverage external resources, a capability particularly relevant in contexts such as Egypt, where institutional support may be inconsistent or limited.

However, other research has suggested that there may be no significant or even an inverse relationship between receiving EE and harboring the intention to pursue entrepreneurship^48,49^.

In the present study, EE is conceptualized primarily as a factor associated with entrepreneurship- and creativity-related outcomes through both direct and indirect pathways. Although moderation effects are not explicitly examined, given the study’s primary focus on mediational mechanisms, prior literature suggests that the influence of EE may extend beyond direct effects and include a broader contextual role. For example, EE may strengthen the relationship between entrepreneurial attitude and intention by transforming favorable attitudes toward entrepreneurship into more concrete entrepreneurial intention^50^. Likewise, EE may reinforce the translation of entrepreneurial intention into creativity by exposing students to creative problem-solving approaches that enhance the creative expression of entrepreneurial goals. Accordingly, moderation is discussed here as a complementary theoretical perspective rather than an empirically tested mechanism within the present model. This study, therefore, proposes that:

##### Hypothesis 1

EE is positively associated with students’ entrepreneurial intention.

#### The relationship between entrepreneurship education, entrepreneurial attitude, and entrepreneurial intention

An individual’s intention to follow an entrepreneurial career is greatly influenced by their attitude toward entrepreneurship. This attitude encompasses the individual’s overall affective and evaluative judgments about entrepreneurship as a career choice^27^. It includes aspects such as the perceived desirability and feasibility of starting a business, as well as the personal appeal of entrepreneurial activities^17^. It is influenced by several factors, including one’s background, experiences, and education. Personal attitude towards entrepreneurship is often characterized by the perceived attractiveness of starting and running a business, the value placed on autonomy and self-realization, and the confidence in one’s ability to succeed as an entrepreneur^28^. According to research, individuals with a favorable orientation toward entrepreneurship are more inclined to identify business opportunities^51^. This positive attitude is also associated with a greater willingness to take risks and resilience to potential setbacks, which are critical traits for entrepreneurial success^52^. Moreover, the literature has begun looking at the mediating role of entrepreneurial attitude in the relationship between EE and entrepreneurial intention. For instance, research by Nowiński et al.^37^ found that personal attitude significantly mediates the association between EE and students’ intention to launch a business. Similarly, Obschonka and Hahn^53^ demonstrated that the way that EE affects intention is mediated in part by changes in students’ attitudes toward entrepreneurship. Therefore, we propose that:

##### Hypothesis 2

EE is positively associated with students’ entrepreneurial attitude.

##### Hypothesis 3

Students’ entrepreneurial attitude is positively associated with their entrepreneurial intention.

##### Hypothesis 4

Entrepreneurial attitude mediates the association between EE and entrepreneurial intention.

### The influence of creativity

#### Entrepreneurship and individual creativity

Creativity plays a pivotal role in entrepreneurship, as it fosters original, practical ideas that are crucial to entrepreneurial success. It is a key aspect of identifying and exploiting opportunities, which are essential in the entrepreneurial process^54^. Similarly, Shaheen et al.^55^ found that creativity has a significant positive effect on entrepreneurial behavior. According to Batchelor and Burch^1^, creativity is inherently attractive to entrepreneurs and is a significant aspect of entrepreneurship. This is supported by a study by Runco and Pritzker^33^, which found a positive association between students’ creativity and their entrepreneurial intentions. The higher the level of student creativity, the stronger the entrepreneurial intention. In addition, individual creativity plays a crucial role in entrepreneurship. For example, in the context of green entrepreneurship, research by Jiang et al.^56^ found that entrepreneurial creativity was favorably connected to green opportunity recognition. This implies that creative entrepreneurs are more likely to recognize and seize opportunities for green entrepreneurship. The study also found that green opportunity recognition is strongly associated with the interaction effect of entrepreneurial inventiveness and green self-identity. This means that when an entrepreneur’s self-identity aligns with green values, their creativity is more likely to lead to the recognition of entrepreneurial opportunities. These findings emphasize the significance of individual creativity in recognizing and seizing entrepreneurial opportunities. They also underscore the need for entrepreneurs to align their self-identity with their entrepreneurial goals to fully leverage their creative abilities. In conclusion, creativity is a critical factor that influences entrepreneurial intention. It is, therefore, necessary to foster creativity in students to enhance EE and promote entrepreneurial intentions. Hence, to explore the interplay between EE, intention, and creativity, the following hypotheses are proposed:

##### Hypothesis 5

EE positively affects the individual creativity of university students.

##### Hypothesis 6

Students’ entrepreneurial intention positively affects their individual creativity.

#### University-level creativity

Anjum et al.^26^ explored how students’ perceptions of university support influence their creativity and entrepreneurial intention. In this context, university support refers to the provision of resources, information, and opportunities that helps students develop entrepreneurial skills and knowledge. The study highlighted that targeted support, such as mentorship in idea generation and business creation, is positively associated with students’ creative tendencies, defined as their ability to generate novel and useful ideas, as well as their intention to engage in entrepreneurial activities. Furthermore, creativity was identified as both an individual trait and a context-sensitive construct, suggesting that supportive classroom environments can provide a platform for students to maximize their creative potential^57^.

EE also plays a significant role in fostering creativity and entrepreneurial intention by enhancing self-efficacy, the belief in one’s ability to achieve specific outcomes. Machali et al.^58^ emphasized that cognitive factors, such as self-efficacy, are influenced by EE that incorporates real-world observations of entrepreneurs. This approach helps students develop decision-making skills and goal-directed behaviors.

Similarly, Aliedan et al.^31^ study demonstrated that university education support affects students’ aspirations for entrepreneurship both directly and indirectly. The study further proposed that the constructs of the TPB, attitude, subjective norms, and perceived behavioral control act as mediators, strengthening the relationship between university support and entrepreneurial intentions. These findings underscore the importance of structured, resource-rich, and behaviorally focused educational interventions in cultivating entrepreneurial mindsets among students. Hence, the following hypotheses are proposed:

##### Hypothesis 7

EE positively affects creativity in the university.

##### Hypothesis 8

Students’ entrepreneurial intention is positively related to creativity in the university context.

### The interplay between EE, entrepreneurial intention, and creativity

Previous literature suggests a strong interplay between EE, entrepreneurial intention, and creativity^31,58^. Students who receive EE are prepared with the abilities and information needed to launch and run enterprises, thereby fostering their entrepreneurial intentions. On the other hand, creativity, which is an inherent aspect of entrepreneurship, enhances these entrepreneurial intentions. According to research by Kusumojanto et al.^45^, EE is crucial for encouraging entrepreneurial intention, and the environment in which EE is provided can significantly influence students’ entrepreneurial intention. This suggests that a supportive environment that encourages creativity and innovation can foster entrepreneurial intention among students. Interestingly, it is found that there is a positive relationship between entrepreneurial intention and creativity. Mukhtar et al.^59^ suggested that more creative people tend to be more inclined to want to start their own business, and an entrepreneurial attitude acts as a mediator in the noteworthy association between EE and entrepreneurial intention. Therefore, creativity and entrepreneurship mutually reinforce each other.

Furthermore, creativity is not only beneficial for students but also for teachers. Teachers’ creativity can influence the quality of EE, which in turn can enhance students’ creativity and entrepreneurial intention^26,60^. This implies a reciprocal relationship among these three elements, in which creativity and EE mutually reinforce each other to enhance entrepreneurial intentions. A similar conclusion was drawn from Shi et al.'s^61^ study, in which creativity strongly mediates the influence of perceived behavioral control on entrepreneurial intention. Moreover, the entrepreneurial environment is also positively associated with students’ entrepreneurial aspirations. A conducive entrepreneurial environment, characterized by supportive policies and a culture that encourages entrepreneurship, can further enhance the effects of EE and creativity on entrepreneurial intention^62^. While the existing literature provides valuable insights into the interplay between EE, entrepreneurial intention, and creativity, there are still some gaps that future research could address. One of the gaps is that most studies focus on the direct effects of EE and creativity on entrepreneurial intention. However, the mediating and moderating effects of the factors have not been thoroughly explored. Hence, this study proposes the following hypotheses:

#### Hypothesis 9

The positive relationship between EE and individual creativity of university students is mediated by students’ entrepreneurial intentions.

#### Hypothesis 10

The positive relationship between EE and creativity in the university context is mediated by students’ entrepreneurial intentions.

#### Hypothesis 11

EE positively influences individual creativity through the sequential mediating roles of entrepreneurial attitude and entrepreneurial intention.

#### Hypothesis 12

EE positively influences university-level creativity through the sequential mediating roles of entrepreneurial attitude and entrepreneurial intention.

## Methods and materials

### Sample techniques

The participants in this study were drawn from undergraduate students enrolled in Tourism and Hotel colleges across six universities in Egypt. We specifically selected these students due to the rising number of entrepreneurial ventures in the food and beverage sector, such as food courts, coffee shops, and food booths throughout Egypt. Many of the proprietors of these establishments are graduates from Tourism and Hotel colleges. Additionally, students in these colleges are required to take more than three courses related to entrepreneurship. Given these factors, we opted to focus on students enrolled in hospitality management programs across these universities as the study’s sample. To conduct the research, we developed an online survey using Google Forms, which targeted university students. After obtaining formal clearances, we distributed the questionnaire to students through their mentors during classes. Mentors shared the survey link via Facebook and WhatsApp groups, encouraging students to further disseminate it among their peers at these universities. A snowball sampling technique was employed to facilitate access to a relevant and engaged sample, particularly through student mentors and peer networks, helping to minimize overlap and encourage serious participation. To enhance the purposiveness of the sample and ensure alignment with the study’s entrepreneurial focus, the selection criteria included: (1) students enrolled in hospitality or tourism management programs who are in their final year (8th level), as they are more likely to be involved in or generate entrepreneurial ideas suitable for implementation after graduation; and (2) prior participation in, or current enrollment in, at least one entrepreneurship-related course, workshop, or program (e.g., business incubators, start-up training, or innovation labs). The total number of students enrolled at all public colleges of tourism and hotels in Egypt is approximately 8230. Following ethical approval from the university, data were collected through an online survey administered via Google Forms between July and September 2025. All participants were required to indicate their informed consent by selecting a checkbox on the online survey before proceeding.

However, we targeted 500 eligible students across the six universities. A total of 337 questionnaires were returned, yielding an overall response rate of 67.4%. After excluding two incomplete or disqualified questionnaires, the final sample used for analysis consisted of 335 valid responses, representing a valid response rate of 67.0% and usable-response rate of 99.4% among the returned questionnaires. Table 1 presents the frequency and percentage distribution of the study participants by gender and university.

**Table 1:** Sociodemographic features of the student population.

Students’ characteristics	Frequency	%
Gender		
Female	152	45.4
Male	183	54.6
University		
Sadat City	72	21.5
Alexandria	65	19.4
Suez Canal	57	17
Fayoum	55	16.4
Helwan	47	14
Luxor	39	11.6

*Source*: Authors’ preparation.

Sample size adequacy for PLS-SEM was assessed primarily through statistical power analysis, as recent methodological recommendations caution against relying solely on the traditional 10-times rule. Following Hair et al.^63^ guidance, sample adequacy was evaluated based on model complexity, particularly the largest number of predictors directed at an endogenous construct. Kock and Hadaya^64^ also note that the ten-times rule may yield inaccurate estimates and recommend power-based approaches for assessing minimum sample size.

In the current study, the most complex endogenous construct had six predictors. Using common power-analysis criteria for multiple regression, medium effect size (f^2^ = 0.15), significance level (α = 0.05), and statistical power (0.80), the required minimum sample size was approximately 98 respondents. Since the final valid sample consisted of 335 respondents, the sample size exceeded this requirement.

As an additional check, the ten-times rule was applied. With six predictors, the largest number of indicators, this rule suggested a minimum sample size of 60 respondents. Therefore, both the power-analysis criterion and the supplementary ten-times rule confirmed that the sample size was adequate for conducting further analysis.

### Measures

The study employed a quantitative approach in line with the research hypotheses. To develop the questionnaire, established and validated scales from existing literature were utilized. The survey was divided into two sections, with the initial part concentrating on evaluating EE, entrepreneurial intention, entrepreneurial attitude, and creativity. The EE scale, comprising 6 items, was sourced from Ouni and Boujelbene^65^. Additionally, the measurement of entrepreneurial intention employed a six-item scale from Do Nguyen and Nguyen^66^, while entrepreneurial attitude was obtained from Liñán and Chen^28^. Creativity assessment involved two scales: individual creativity (3 items) and university-level creativity (3 items), adapted from Wang et al.^21^. All indicators for the research variables were scored on a 7-point Likert scale, ranging from 1 to 7 (ranging from strongly disagree to strongly agree). The subsequent section of the survey collected demographic information from participants. Some items have been adapted to align with the objectives of the research. For example, the statement 'This course provides the required knowledge of entrepreneurship’ was modified to 'I studied a course (or courses) that provided me with the necessary knowledge of entrepreneurship. To ensure clarity and encourage participation, the questionnaire was translated from English to Arabic. Before distribution, the contextual validity of the Arabic version was confirmed through back-translation into English. A pilot study involving 30 student participants was conducted to evaluate the reliability and validity of the questionnaire, with input from three experts in entrepreneurship within the hospitality and tourism sector, leading to slight adjustments in the Arabic version.

### Common-method bias (CMB)

Given the cross-sectional and self-reported nature of the data, potential common method bias was addressed through both procedural and statistical remedies. Procedurally, respondents were assured of anonymity and confidentiality, and the questionnaire was designed with clear instructions and well-structured items to reduce ambiguity and minimize response bias^67^. Statistically, Harman’s single-factor test was conducted using principal component analysis. The results showed that the first unrotated factor accounted for 46.70% of the total variance, which is below the commonly accepted 50% threshold. This indicates that no single factor dominated the data’s variance structure, suggesting that common method bias was unlikely to pose a serious threat to the validity of the findings.

Additionally, an alternative model incorporating a randomly generated marker variable was tested to further examine potential common method bias and assess whether it could have distorted the study results. The inclusion of this variable did not produce substantial changes in the estimated relationships among the main research constructs, providing further evidence that common method bias was not a major concern.

Finally, collinearity diagnostics were conducted to assess potential multicollinearity among the constructs. The maximum variance inflation factor was 3.4, and all VIF values remained below the recommended threshold of 5^68^. These findings indicate that multicollinearity was not a significant issue in the model. These findings provide sufficient evidence that neither common method bias nor multicollinearity substantially biased the estimated relationships among the research constructs.

### Data analysis techniques

To address the research questions, we used the PLS-SEM approach for data analysis. PLS is well recognized for its efficacy in estimating path coefficients in structural models, especially in hospitality and tourism research. Accordingly, to test the research hypotheses, SmartPLS-SEM version 4 was employed. The assessment of the theoretical model involved a two-step procedure using the software, initially scrutinizing the outer model and subsequently evaluating the structural model.

### The outer model

As shown in Table 2, the Composite Reliability (CR) for all latent variables (LVs) in the measurement model exceeded the 0.7 threshold, as recommended by Hair et al.^68^. This suggests that the measurement model demonstrated internal consistency and reliability. Additionally, all item loadings surpassed the recommended value of 0.7, while construct CR values were higher than 0.7, and Average Variance Extracted (AVE) values surpassed the threshold of 0.5 according to Hair et al.^68^, as shown in Table 2, confirming the establishment of convergent validity. As depicted in Table 3, discriminant validity was affirmed as the Heterotrait-Monotrait (HTMT) values did not exceed the threshold of 0.85^68,69^, and all construct correlations were lower than the square root of AVE for their respective constructs, establishing the Fornell-Larcker criterion^70^, see Table 4. The highest Variance Inflation Factor (VIF) was 3.4, and all indicators were below the threshold of 5, confirming the absence of multicollinearity among the constructs, as outlined by Ringle et al.^71^. These findings collectively support the reliability and validity of the scales.

**Table 2:** Results of validity, reliability, and descriptive frequencies.

	Loadings
Entrepreneurship education (EE) (α = 0.880, CR = 0.910, AVE = 0.629)	
EE1: The EE offered by the university/college encourages me to develop creative ideas for being an entrepreneur	0.829
EE2: I studied a course (or courses) that provided me with the necessary knowledge of entrepreneurship	0.823
EE3: This course (s) developed my entrepreneurial skills and abilities	0.872
EE4: This course (s) provides students with relevant entrepreneurship material and teaches them how to start a business	0.773
EE5: I thought that entrepreneurship could be enlarged through education	0.701
EE6: I think that EE in the faculty helps students to be an entrepreneur	0.757
Entrepreneurial intention (α = 0.914, CR = 0.933, AVE = 0.700)	
EI1: I’m ready to do anything to be an entrepreneur	0.742
EI2: My professional goal is to become an entrepreneur	0.842
EI3: I will make every effort to start and run my firm	0.869
EI4: I’m determined to create a firm in the future	0.885
EI5: I have very seriously thought about starting a firm	0.827
EI6: I’ve got the firm intention to start a firm someday	0.848
Entrepreneurial attitude (α = 0.885, CR = 0.916, AVE = 0.686)	
EA1: Being an entrepreneur implies more advantages than disadvantages to me	0.732
EA2: A career as an entrepreneur is attractive to me	0.865
EA3: If I had the opportunity and resources, I’d like to start a firm	0.788
EA4: Being an entrepreneur would entail great satisfaction for me	0.877
EA5: Among various options, I would rather be an entrepreneur	0.870
Individual creativity (α = 0.788, CR = 0.876, AVE = 0.701)	
IC1: I think I am a very creative person	0.844
IC2: I like to try novel things despite the risk of failure	0.824
IC3: I can easily think of a lot of different and useful ideas	0.844
University-level creativity (α = 0.885, CR = 0.929, AVE = 0.813)	
UC1: I learned that there is more than one solution to solve problems in your university	0.881
UC2: I learned to examine old problems in new ways at your university	0.922
UC3: The faculty encourages students to produce new and useful ideas in your university	0.902

*Source*: Authors’ preparation.

**Table 3:** Heterotrait-Monotrait ratio (HTMT) – matrix.

No	Variable	1	2	3	4	5
1	Entrepreneurship education					
2	Entrepreneurial intention	0.602				
3	Entrepreneurial attitude	0.631	0.882			
4	Individual creativity	0.526	0.681	0.727		
5	University-level creativity	0.736	0.518	0.597	0.655	

*Source*: Authors’ preparation.

**Table 4:** Fornell-Larcker criterion.

	Construct	1	2	3	4	5
1	Entrepreneurship education	**0.793**				
2	Entrepreneurial intention	0.541	**0.837**			
3	Entrepreneurial attitude	0.557	0.804	**0.828**		
4	Individual creativity	0.442	0.583	0.611	**0.837**	
5	University-level creativity	0.654	0.469	0.525	0.548	0.902

Significant values are in [bold]

*Source*: Authors’ preparation.

### The structural model

The evaluation of the structural model involves examining R2 values, p-values, effect sizes (f2), and the significance of path coefficients (β). The model’s results showed R2 values of 31%, 36.2%, 44.7%, and 65.9% for all dependent variables, entrepreneurial attitude, individual creativity, university-level creativity, and entrepreneurial intention, respectively, indicating that these variables explain approximately 31% to 66% of the variance. This reflects a consecutive explanatory power, as outlined by Chin^72^. Additionally, Table 5 displays both β and P-values, confirming the statistical significance between the model variables. Furthermore, the model’s predictive relevance (Q2) was assessed, and as the Q2 values surpassed zero (e.g., 0.181 for Individual creativity, 0.285 for entrepreneurship intention, 0.300 for entrepreneurial attitude, and 0.415 for university-level creativity), the current study’s model exhibits strong predictive capability following the criteria set by Hair et al.^68^. Lastly, the effect size (f2) of the research model was scrutinized using Cohen’s criteria, revealing f-square effect sizes ranging from 0.031 to 1.07283, indicating weak, medium, and strong effect sizes among the research variables.

**Table 5:** Summary of the research hypotheses.

H	Path	Β	t-value	*p*-value	95% CI [LLCI, ULCI]	Decision
H1	EE → Entrepreneurial intention	0.135**	2.622	0.009	[0.037, 0.236]	Supported
H2	EE → Entrepreneurial attitude	0.557***	10.103	*p* < 0.001	[0.454, 0.666]	Supported
H3	Entrepreneurial attitude → Entrepreneurial intention	0.728***	14.541	*p* < 0.001	[0.625, 0.821]	Supported
H5	EE → Individual creativity	0.179**	2.631	0.008	[0.042, 0.307]	Supported
H6	Entrepreneurial intention → Individual creativity	0.486***	7.151	*p* < 0.001	[0.349, 0.619]	Supported
H7	EE → University-level creativity	0.566***	10.072	*p* < 0.001	[0.456, 0.675]	Supported
H8	Entrepreneurial intention → University- level creativity	0.163*	2.150	0.031	[0.016, 0.310]	Supported
H4	EE → Entrepreneurial attitude → Entrepreneurial intention	0.406***	7.598	*p* < 0.001	[0.309, 0.519]	Partial mediation
H9	EE → Entrepreneurial intention → Individual creativity	0.066*	2.506	0.012	[0.016, 0.120]	Partial mediation
H10	EE → Entrepreneurial intention → University- level creativity	0.022	1.590	0.112	[-0.005, 0.054]	Rejected
H11	EE → Entrepreneurial attitude → Entrepreneurial intention → Individual creativity	0.197***	4.612	*p* < 0.001	[0.121, 0.289]	Supported
H12	EE → Entrepreneurial attitude → Entrepreneurial intention → University- level creativity	0.066*	2.072	0.038	[0.008, 0.135]	Supported

*Source*: Author’s preparation.

## Testing the research hypotheses

This section presents the results of the hypothesis testing, focusing on the relationships between EE, EA, intentions, and creativity. The analysis evaluates both direct and indirect effects, highlighting the pathways through which EE influences individual and university-level creativity. The results are systematically outlined, emphasizing significant findings and supported hypotheses. These findings are integral to understanding EE’s role in fostering entrepreneurial mindsets and creative capabilities among students.

### Results of the direct relationships

**Fig. 2:**
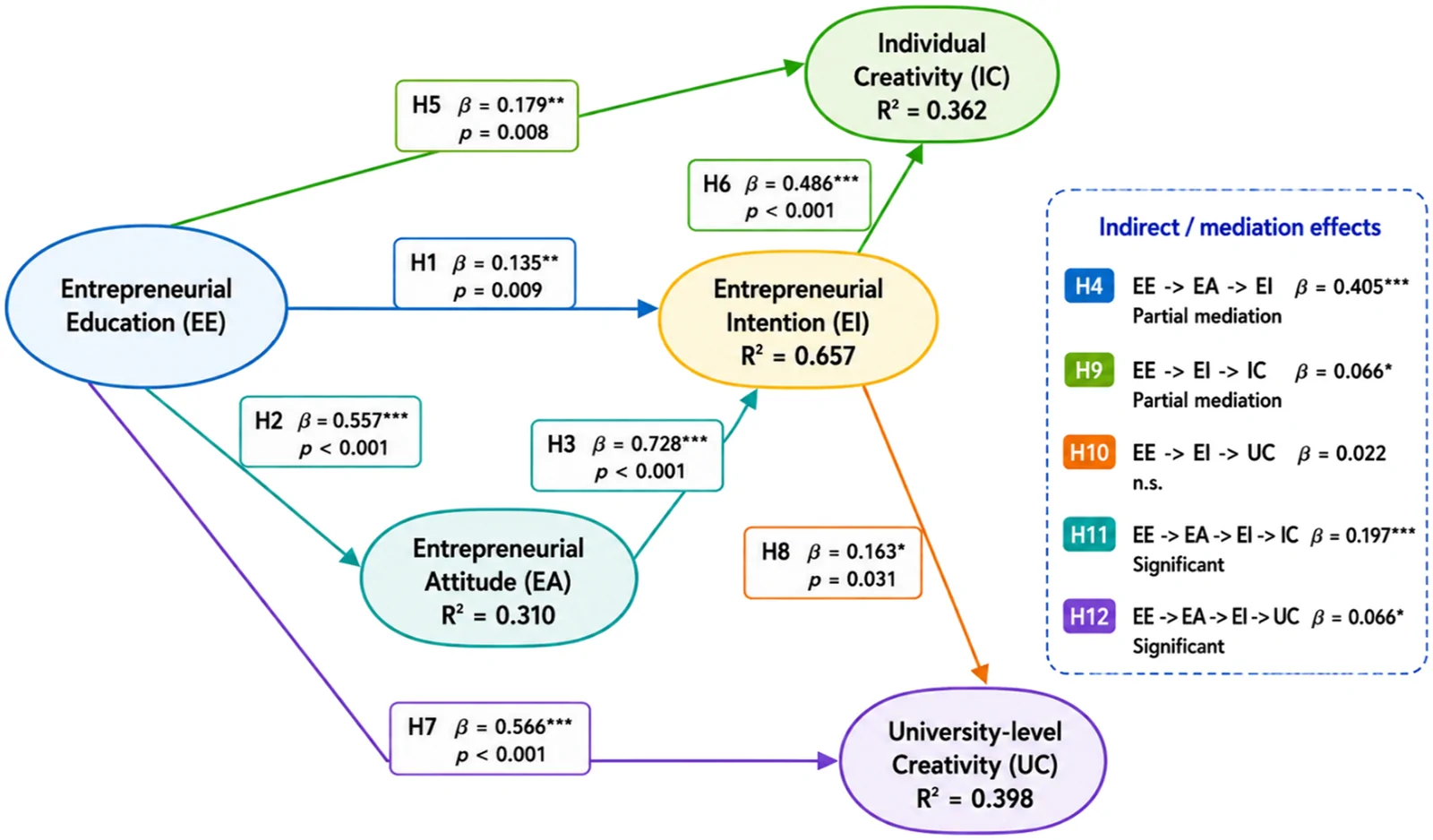
The structural modeling analysis. *p* < 0.05*; *p* < 0.01**; *p* < 0.001***.

To test the research hypotheses, a bootstrapping procedure with 5000 resamples was conducted to estimate the path coefficients, t-values, p-values, and 95% bootstrapped confidence intervals. As illustrated in Table 5 and Fig. 2, the results of the direct relationships show that EE had a positive and significant effect on entrepreneurial intention (β = 0.135, t = 2.622, *p* = 0.009), entrepreneurial attitude (β = 0.557, t = 10.103, *p* < 0.001), individual creativity (β = 0.179, t = 2.631, *p* = 0.008), and university- level creativity (β = 0.566, t = 10.072, *p* < 0.001). Therefore, H1, H2, H5, and H7 were supported, respectively. Finally, entrepreneurial attitude had a positive and significant effect on entrepreneurial intention (β = 0.728, t = 14.541, *p* < 0.001), supporting H3. Similarly, entrepreneurial intention had a positive and significant effect on both individual creativity (β = 0.486, t = 7.151, *p* < 0.001) and university- level creativity (β = 0.163, t = 2.150, *p* = 0.031), supporting H6 and H8, respectively.

### Indirect relationship results

We utilized a bootstrapping technique with 5000 samples to compute the Confidence Interval (CI), t-values, p-values, and path coefficients. When examining the mediating effects, we followed the two approaches recommended by Preacher and Hayes^73^, specifically utilizing bootstrapping for both the indirect effect and the confidence interval. The findings of the bootstrapping technique confirmed the mediating effect of entrepreneurial attitude on the relationship between EE and entrepreneurial intention (β = 0.406, t = 7.598, *p* < 0.001), with a 95% confidence interval (LLCI = 0.040, ULCI = 0.250). Not surprisingly, the results also confirmed the mediating role of students’ entrepreneurial intention in the relationship between EE and individual creativity of university students (β = 0.066, t = 2.506, *p* < 0.05), with an interval (LLCI = 0.016, ULCI = 0.120). As the T-values for both mediators (i.e., entrepreneurial attitude and students’ entrepreneurial intention) exceeded 1.96, and the Bootstrapped Confidence Interval (BCI) did not include zero, this indicates partial mediation, as both the direct and indirect effects remained significant. Thus, H4 and H9 are supported, respectively. Unexpectedly, the findings showed that students’ entrepreneurial intention does not mediate the relationship between EE and university-level creativity (β = 0.022, t = 1.59, *p* > 0.05), with an interval (LLCI = 0.001, ULCI = 0.054). Hence, H10 is rejected, see Table 5.

The results confirmed the sequential mediating role of entrepreneurial attitude and entrepreneurial intention in the relationship between EE and individual creativity. Specifically, EE had a significant indirect effect on individual creativity through entrepreneurial attitude and entrepreneurial intention (β = 0.197, t = 4.612, *p* < 0.001, LL_CI_ = 0.121, UL_CI_ = 0.289). Since the confidence interval did not include zero, H11 was supported.

Similarly, the results showed that EE had a significant sequential indirect effect on university- level creativity through entrepreneurial attitude and entrepreneurial intention (β = 0.066, t = 2.072, *p* = 0.038, LL_CI_ = 0.008, UL_CI_ = 0.135). As the bootstrapped confidence interval did not include zero, H12 was supported.

These findings indicate that EE enhances students’ entrepreneurial attitude, which strengthens their entrepreneurial intention and subsequently improves both individual creativity and university-level creativity. Since the corresponding direct effects remained significant, the results suggest partial sequential mediation.

## Discussions

Our findings affirm that EE has a significant positive effect on students’ aspirations to launch ventures, corroborating prior studies^74–76^. This influence manifests as a pivotal driver of entrepreneurial intention, characterized by the desire and determination to engage in entrepreneurial activity. However, our results also contrast with earlier research^77,78^, which argues that EE does not necessarily lead to higher entrepreneurial intentions. These discrepancies can be attributed to contextual differences, such as variations in program structure, pedagogical approaches, and the socio-economic environment in which the education is delivered.

In the Egyptian context, the integration of EE into university curricula has proven instrumental in enhancing students’ entrepreneurial attitude, intentions, and creativity. This aligns with findings from Do Nguyen and Nguyen^66^, who highlight that EE not only equips students with theoretical knowledge but also prepares them for real-world challenges, fostering a mindset of resilience and innovation. By transforming students from passive learners into active creators, EE shifts their role from job seekers to proactive job creators, contributing to sustainable socioeconomic development. This transformation is critical in addressing Egypt’s high rates of graduate unemployment while aligning educational outcomes with the nation’s economic priorities.

In the Egyptian context, this relationship is shaped by unique socio-economic factors, including a high demand for entrepreneurial solutions to unemployment and economic stagnation. The structured integration of EE within Egyptian universities highlights its role in supporting students’ transition from job seekers to potential job creators. This supports broader socioeconomic development goals, particularly in a context where opportunities for traditional employment may be limited.

The economic context of Egypt, characterized by a youthful population with untapped entrepreneurial potential, magnifies the importance of embedding EE within higher education. These findings resonate with studies conducted in comparable service-driven and emerging economies. For instance, in countries like India and Indonesia, where the job market faces similar structural constraints, EE has been observed to play a pivotal role in shifting attitudes and intentions^15,79^.

The results of this study offer meaningful extensions to the TPB^10^, particularly in a non-Western, developing context. The significant relationships identified between EE, entrepreneurial attitude, and intentions support TPB’s central premise that behavioral intention is shaped by evaluative beliefs^27,28^. However, this study also reveals that entrepreneurial intention has a mediating role between EE and individual creativity, suggesting that TPB may have a broader application when creativity is treated as a behavioral outcome, a contribution that goes beyond its traditional use in intention-behavior models.

In terms of the CTC^11^, the findings align with the notion that domain-relevant skills (such as those developed through EE) and intrinsic motivation (captured through entrepreneurial intention) are critical in fostering creativity. The study extends this theory by demonstrating that EE not only supports individual creativity but also university-level creativity, although with limited mediation through intention. This implies that organizational creativity may depend more on systemic institutional support than on individual cognition, suggesting a layered application of Amabile’s model in university contexts within developing countries.

Moreover, the results substantiate key insights from Social Cognitive Theory^34^, particularly regarding the interplay of environmental support, self-belief, and behavior. The presence of entrepreneurship clubs, role models, and innovation hubs within universities supports the idea that contextual reinforcements enhance self-efficacy, indirectly nurturing entrepreneurial intention and creative outcomes. However, the relatively weak mediation effect of entrepreneurial intention on UC suggests that institutional creativity may require more than individual self-efficacy, such as policy-level interventions and faculty engagement.

Finally, this study contributes to the Entrepreneurial Event Model^35^ by demonstrating that EE helps improve perceptions of the feasibility and desirability of entrepreneurship, especially among final-year students in a developing context. The strong predictive relationships between EE and entrepreneurial intention confirm that such perceptions can indeed trigger intent, but only when EE is structured, localized, and relevant to students’ real-world experiences.

Our study also underscores the role of cultural factors. Egyptian society has traditionally emphasized stability through government or corporate employment. However, EE is challenging this paradigm by fostering a mindset that values creativity, risk-taking, and innovation. This aligns with studies from other developing economies, such as Turkey and South Africa, where EE has cultivated favorable attitudes toward entrepreneurship as a viable and rewarding career path^13,80^.

Entrepreneurial attitude serves as a vital mediator in the relationship between EE and entrepreneurial intentions, as supported by prior studies^13,80,81^. From the perspective of the TPB, attitudes toward behavior are key predictors of intention. EE shapes these attitudes by nurturing creativity, encouraging risk-taking, and fostering independence, traits that align with the positive evaluative beliefs emphasized in TPB. These characteristics are essential for cultivating a proactive entrepreneurial disposition, which, in turn, strengthens the intention to engage in entrepreneurial behavior.

Furthermore, an entrepreneurial attitude embodies the emotional and motivational elements that catalyze action^82^, resonating with the TPB’s assertion that favorable attitudes increase the likelihood of intention formation. Al Halbusi et al.^83^ emphasize that external influences such as role models and social media exposure can further enhance these attitudes, functioning as part of the social normative pressures within TPB that shape behavioral intentions. These insights reinforce the importance of embedding innovative, contextually relevant EE programs, particularly in Egypt, to enhance students’ entrepreneurial mindsets and intention pathways in accordance with the TPB framework.

Universities play a central role in this process by extending their influence beyond traditional classrooms. As suggested by Hameed and Irfan^84^ and Hassan et al.^85^, universities must foster an ecosystem that encourages risk-taking, nurtures creativity, and supports entrepreneurial activities. This holistic approach includes offering practical training opportunities, entrepreneurial mentorship, and access to resources that prepare students for the complexities of entrepreneurship. For example, Zine et al.^86^ emphasize the importance of entrepreneurial training to bridge the gap between academic knowledge and practical application. Utami^87^ further argues that experiential learning opportunities can foster students’ enthusiasm for entrepreneurship, thereby supporting the relevance and effectiveness of educational programs.

Our research also highlights a strong correlation between entrepreneurial intention and individual creativity, aligning with existing studies^61,88^. Creative individuals often display resilience and a positive mindset, enabling them to navigate the uncertainties inherent in entrepreneurial ventures. Additionally, entrepreneurial aspirations enhance innovative thinking and problem-solving abilities, which are critical for identifying and seizing market opportunities^89^. This suggests that entrepreneurial intention is not only a predictor of entrepreneurial behavior but also a catalyst for individual creativity, as entrepreneurial individuals actively seek novel solutions and approaches.

However, our findings extend the scope of prior research by examining creativity at the institutional level. While EE fosters individual creativity, its influence on university-level creativity is less direct. Institutional creativity requires collaborative environments and systemic support to flourish. Simonton^90^ and Anjum et al.^26^ emphasize that universities and governments must collaborate to establish ecosystems that stimulate innovation. This includes organizing entrepreneurship competitions, providing funding for student ventures, and offering access to incubation centers. In this context, the role of EE in Egypt extends beyond teaching entrepreneurial skills; it must also instill a culture of creativity and innovation within academic institutions.

A particularly notable aspect of our findings is the mediated relationship between EE, entrepreneurial intentions, and creativity. While previous studies^15,79^ have established the mediating role of attitude in influencing entrepreneurial intentions, our research demonstrates that entrepreneurial intention also mediates the relationship between EE and individual creativity. This is consistent with the findings of Al Halbusi et al.^91^, who emphasize the interconnectedness of intentions and creativity, particularly in digitally driven entrepreneurial ecosystems. However, the relationship between EE and university-level creativity remains less mediated by entrepreneurial intentions, suggesting that fostering institutional creativity requires additional mechanisms beyond individual aspirations.

H10 is not supported, indicating that entrepreneurial intention does not mediate the relationship between EE and university-level creativity. Contrary to the results found in the Chinese setting (Wang et al.^21^), university-based creativity in Egypt appears to be influenced more by factors such as institutional infrastructure, mentorship programs, innovation clusters, sources of funding, and organizational culture, rather than by students’ entrepreneurial intentions. In an environment of scarce resources, it is plausible that institutional novelty is derived more from systemic support than from individual impetus. This finding warrants further examination.

Although EE exerts a significant positive direct effect on university-level creativity (H7: β = 0.566, *p* < 0.001), this influence does not operate through students’ entrepreneurial intentions. Three possible explanations are proposed.

First, the distinction between institutional and individual-level mechanisms may account for this outcome. university-level creativity was conceptualized as students’ perceptions of their university as an environment that encourages novel thinking, problem-solving, and creativity (e.g., “The instructors urge me to come up with new and useful ideas”). Unlike individual creativity , which reflects an internal cognitive capability, university-level creativity represents an external and structural institutional characteristic. As an intrapersonal factor, entrepreneurial intention may therefore play a limited role in shaping perceptions of institutional climate. Instead, university-level creativity is more likely to be influenced by system-level factors such as curriculum design, faculty training, availability of innovation labs, intellectual property (IP) policies, and administrative support for student entrepreneurial initiatives. This distinction is consistent with Simonton^90^, who suggests that organizational creativity is shaped more strongly by environmental press, external resources, and contextual pressures than by individual characteristics.

Second, this finding differs from the results reported by Wang et al. in China, where a study of 1873 university students found that the relationship between EE and creativity was positively and significantly mediated by entrepreneurial intention (referred to as entrepreneurial inspiration in their framework) at both individual and institutional levels. One possible explanation for this discrepancy lies in differences in entrepreneurial ecosystems. China benefits from comparatively strong institutional support for entrepreneurship, including government-sponsored incubators, university–industry collaborations, streamlined business registration systems, and a national innovation-oriented policy environment^92^. In such contexts, entrepreneurial intention may translate more effectively into perceptions of institutional creativity because supporting structures reinforce entrepreneurial engagement.

In contrast, Egypt’s entrepreneurial environment remains relatively constrained, with challenges such as limited access to funding, bureaucratic barriers, and risk-averse societal norms^9^. In such contexts, students’ entrepreneurial intention alone may be insufficient to influence broader perceptions of institutional creativity. Even highly motivated students may have limited capacity to affect university-level innovation in the absence of structural support mechanisms such as entrepreneurship centers, industry partnerships, and updated curricula. Therefore, the mediating pathway appears to depend on both individual initiative and institutional readiness, conditions that may not fully coexist in resource-constrained settings.

Third, temporal and feedback effects may further explain the non-significant mediation of H10. University-level creativity may evolve not only as an outcome but also as a contextual antecedent of entrepreneurial intention, particularly in resource-constrained environments. Students who perceive their university as supportive of creativity may gradually develop stronger entrepreneurial intention over time. However, the cross-sectional nature of the data does not allow these temporal dynamics to be fully disentangled. Future longitudinal research could investigate whether entrepreneurial intention subsequently influences perceptions of university-level creativity through sustained engagement with institutional resources, such as participation in student organizations and innovation activities.

In conclusion, the rejection of H10 highlights an important boundary condition of the theoretical model. While entrepreneurial intention mediates the relationship between EE and individual creativity (as supported in H9), it does not exert the same mediating effect at the university level. This suggests that fostering institutional creativity requires more than cultivating entrepreneurial mindsets at the individual level; it also requires structural and organizational support, which has important implications for policymakers and educators in developing economies.

A further result of this study is that the sequential mediating effect of entrepreneurial attitude and entrepreneurial intention was corroborated in the path from EE to individual and university-level creativity (H11 and H12). These results imply that EE enhances creativity not just by acquiring knowledge itself; rather, it occurs through a sequential psychological process where students first form positive attitudes toward entrepreneurship, which later solidify into entrepreneurial intention and finally lead to creative outcomes. This result contributes to the TPB by expanding both entrepreneurial attitude and intention as determinants of, and as psychological paths through which, creativity could be nurtured in educational contexts, not only to predict entrepreneurial behavior in educational contexts but also in the educational context itself. It is also suggested that the development of creativity in EE is influenced, at least in part, by motivational and cognitive changes rather than purely technical entrepreneurial skills. In fact, this sequential process may be particularly relevant in the context of Egyptian higher education, where students’ perceptions of self-employment, risk-taking, and innovation may be progressively shaped through EE, thereby boosting entrepreneurial mindsets and creative capacities.

Our results also resonate with the broader discourse on the role of EE in fostering innovation. Edwards-Schachter et al.^32^, Hamidi et al.^93^, and Wang et al.^21^ all highlight how exposure to EE enhances students’ capacity to generate groundbreaking ideas. In this study, we contend that creativity is both an outcome and a critical enabler of entrepreneurial intention. Aspiring entrepreneurs, equipped with the knowledge and skills gained through EE, are better prepared to develop innovative products and services that thrive in competitive markets. This underscores the value of integrating creativity-focused modules into EE programs.

Recognizing these dynamics, Egyptian universities must prioritize developing entrepreneurial ecosystems that empower students to translate creative ideas into actionable ventures. This requires a dual focus on cultivating individual creativity and fostering an environment conducive to innovation. Ali and Abou^94^ argue that a combination of personal and environmental factors is essential for enhancing innovation within academic institutions. Therefore, collaboration between universities and policymakers is crucial for sustaining this momentum. By providing financial incentives, mentorship programs, and infrastructural support, the government can help universities create an enabling environment for entrepreneurship.

In conclusion, our findings emphasize the transformative potential of EE in fostering entrepreneurial attitude, intentions, and creativity. While individual creativity is significantly influenced by EE and mediated by entrepreneurial intentions, institutional creativity requires targeted support to thrive. By addressing both dimensions, EE can serve as a cornerstone for developing innovative and entrepreneurial talent in Egypt, ultimately contributing to the country’s sustainable development. These insights underscore the need for universities to adopt a comprehensive approach that integrates entrepreneurial training, fosters innovation, and prepares students to navigate the complexities of entrepreneurship in emerging economies.

Overall, this study underscores the importance of contextualizing mainstream theories of EE when applied in emerging economies. While foundational models like TPB and CTC remain relevant, their predictive power increases when paired with cultural, institutional, and economic considerations, as informed by SCT and EEM. These findings advocate for a more integrated, flexible use of theory in EE research, one that reflects the realities of developing-world educational and entrepreneurial ecosystems.

## Implications

### Practical implications

This study offers three key practical implications. By examining the relationships between EE, entrepreneurial attitude, entrepreneurial intentions, individual creativity, and university-level creativity, the findings underscore the multifaceted nature of entrepreneurship.

Firstly, the results emphasize the importance of integrating EE into university curricula to foster positive attitudes toward entrepreneurship. As Kuratko^95^ highlights, structured entrepreneurship programs may play an important role in shaping student mindsets. These programs can cultivate entrepreneurial intention by enhancing creativity and promoting innovative thinking. Universities should actively foster an environment that supports creativity at both individual and institutional levels. A creative university atmosphere plays a vital role in shaping entrepreneurial mindsets, driving innovative thought, and encouraging entrepreneurial activity. For example, institutions like Stanford University and the Massachusetts Institute of Technology serve as benchmarks, demonstrating how a strong emphasis on innovation can yield numerous successful startups. Inspired by these models, the Egyptian Ministry of Higher Education should consider incorporating similar strategies into the nation’s entrepreneurship roadmap. Practical steps might include establishing alumni mentorship programs, fostering industry partnerships, providing investment funds, and encouraging participation in entrepreneurship-focused contests.

Secondly, policymakers and educators should collaborate to design and implement educational programs that prioritize the development of creative skills, entrepreneurial attitude, and intentions. Experiential learning opportunities, such as internships, workshops, and competitions, are particularly effective, as they challenge students to apply their knowledge in real-world entrepreneurial scenarios. Activating entrepreneurship offices in Egyptian universities is another crucial step. These offices can provide guidance and resources to aspiring student entrepreneurs, supporting them in initiating and developing their entrepreneurial ventures.

Finally, the study reveals that fostering an entrepreneurial ecosystem within educational institutions significantly enhances students’ readiness to embark on entrepreneurial ventures. This readiness translates into increased startup formation, job creation, and economic diversification. These broader societal benefits underscore the importance of investing in EE and nurturing a creative university culture, not only for individual student success but also for national and economic advancement.

### Theoretical implications

Theoretically, this study offers valuable contributions to entrepreneurship research by reinforcing and extending established frameworks that have been fully discussed in the literature review. The findings support the TPB, which posits that an individual’s behavioral intention is the most immediate predictor of actual behavior. In this study, TPB provides a useful lens to understand how EE influences entrepreneurial intention through two key mediators: entrepreneurial attitude and perceived behavioral control. These results underscore the role of educational experiences in shaping students’ cognitive and motivational readiness for entrepreneurial action.

Moreover, the findings highlight the role of individual creativity as a significant antecedent to entrepreneurial intention, in line with CTC, which was also elaborated in the theoretical framework. This theory explains how task motivation, creativity-relevant skills, and domain-relevant knowledge interact to influence creative output, suggesting that fostering creativity should be a central goal of EE. By confirming the relevance of these theories within the context of hospitality students, this study not only supports but also extends their applicability to educational settings in emerging markets. The implication is that to boost students’ self-efficacy and entrepreneurial intentions, institutions should foster cultures that value creativity and innovation. The study also touches on the Entrepreneurial Event Model^35^, which postulates that perceptions of feasibility and desirability play a role in the choice to launch a new business. The results suggest that EE can boost entrepreneurial intention by raising the perceived feasibility and desirability of entrepreneurial professions.

In summary, the theoretical implications of this study suggest that the TPB provides a robust framework for understanding the association between EE and entrepreneurial intentions. However, the findings also indicate that integrating additional theoretical perspectives, such as the CTC, Social Cognitive Theory, and the Entrepreneurial Event Model, can offer a more comprehensive understanding of the factors that shape entrepreneurial behavior.

## Limitations and future research directions

Despite the valuable contributions of this study, several limitations should be acknowledged, which also provide directions for future research. First, the generalizability of the findings is constrained by the use of snowball sampling and the possibility of self-selection bias. Since participation was voluntary and facilitated through mentor and peer networks, the sample may not fully represent the broader population of Tourism and Hotel college students in Egypt.

Another important limitation relates to the cross-sectional nature of the study, which prevents the examination of how entrepreneurial intention evolves into actual entrepreneurial behavior over time. Although the Theory of Planned Behavior (TPB) identifies intention as the closest predictor of behavior, previous research has consistently highlighted the existence of an intention–behavior gap, where entrepreneurial intention does not necessarily lead to venture creation due to contextual and personal barriers. A recent systematic bibliometric analysis by Zhuang and Sun reconfirmed this persistent divide and emphasized the role of both individual-level factors, such as education and self-efficacy, and environmental factors, including university support and cultural norms, in shaping the transition from entrepreneurial intention to entrepreneurial action.

Within the Egyptian hospitality context, this gap may be particularly significant because of weak entrepreneurial infrastructure, cultural risk aversion, bureaucratic constraints, and limited access to seed funding. Therefore, future studies should adopt longitudinal research designs to track the development of entrepreneurial intention and entrepreneurial outcomes over time. Such studies could measure entrepreneurial intention before and after EE programs and include follow-up assessments after six and twelve months. In addition, future research should examine actual entrepreneurial behaviors, including venture creation, business registration, first sales, and employee hiring. Longitudinal approaches would provide deeper insights into the extent to which EE helps students translate intentions into action and influences their long-term career trajectories and entrepreneurial outcomes.

Future research should also extend beyond a single national context by conducting comparative studies across different cultural and educational systems. Such comparisons would enhance understanding of how contextual differences influence entrepreneurial attitude, intentions, and creativity outcomes. Furthermore, additional investigation is needed into the role of university–industry collaboration, particularly regarding experiential learning opportunities and opportunity recognition among hospitality students.

Finally, given the rapid advancement of digital technologies in entrepreneurship and education, future studies should explore how digital tools, online platforms, and technology-mediated learning environments influence the effectiveness of EE. Particular attention should be given to their potential role in enhancing creativity development and entrepreneurial intention formation among university students.

## Data Availability

The datasets generated and/or analyzed during the current study are not publicly available due to privacy concerns; however, the data presented in this study are available upon request from the corresponding authors.
